# A Longitudinal Peri-Implant Diaphyseal Fracture Around a Locked Humeral Nail: A Case Report

**DOI:** 10.3390/reports8020089

**Published:** 2025-06-05

**Authors:** Ana del Potro Jareño, Alfonso González Menocal, Ana Antonia Couceiro Laredo, Laura Conde Ruiz, Daniel López Dorado

**Affiliations:** Hospital Universitario Infanta Elena, 28342 Valdemoro, Spain; alfonso.gmenocal@gmail.com (A.G.M.); ana.couceirolaredo@gmail.com (A.A.C.L.); lauraconderuiz@gmail.com (L.C.R.); lofishdorado@gmail.com (D.L.D.)

**Keywords:** periprosthetic fractures, fractures, bone, humerus, fracture fixation, intramedullary, internal

## Abstract

**Background and Clinical Significance**: Non-prosthetic peri-implant fractures (NPPIFs) are rare injuries occurring around internal fixation devices, and are distinct from periprosthetic fractures. While most studies focus on the femur, humeral NPPIFs remain poorly documented. This case illustrates a complex humeral NPPIF and highlights key surgical considerations. **Case Presentation**: A 62-year-old woman presented with a spiral humeral shaft fracture (AO 12B2) after a fall. Following closed reduction and antegrade intramedullary nailing, an intraoperative peri-implant fracture occurred at the distal interlocking screw. CT imaging revealed a complex fracture extending from the lateral condyle to the proximal humerus. Treatment included implant removal and open reduction with dual plate fixation—lateral distal and helically contoured proximal plates—plus cerclage bands and antibiotic-loaded beads. Recovery was uneventful, with a full range of motion achieved at six months. At one year, the DASH score and MEPS were 86 and 75, respectively. **Conclusions**: Humeral NPPIFs are challenging and require individualized, biomechanically sound strategies. This case reinforces the importance of intraoperative assessment and careful implant selection in humeral fracture management.

## 1. Introduction and Clinical Significance

The term peri-implant fracture refers to a loss of bone continuity around a surgical implant. Such fractures may occur around a replacement prosthesis (periprosthetic fractures) or around rods, nails or plates (non-prosthetic peri-implant fractures [NPPIFs]) [1,2]. Although the former have spawned a significant amount of literature, it was not until recently that NPPIFs were recognized as a separate clinical entity, with all their corresponding conceptual and practical implications [1,3].

As a matter of fact, most of the data on NPPIFs published in the literature are limited to small case series dedicated to complications in the femur. For that reason, some authors have emphasized the need to investigate and document NPPIFs occurring in other, less studied, anatomical regions such as the tibia, the forearm and the humerus [1]. The present case report, which deals with an NPPIF associated with an intramedullary humeral nail, aims to address that gap in the literature.

## 2. Case Presentation

**Figure 1:**
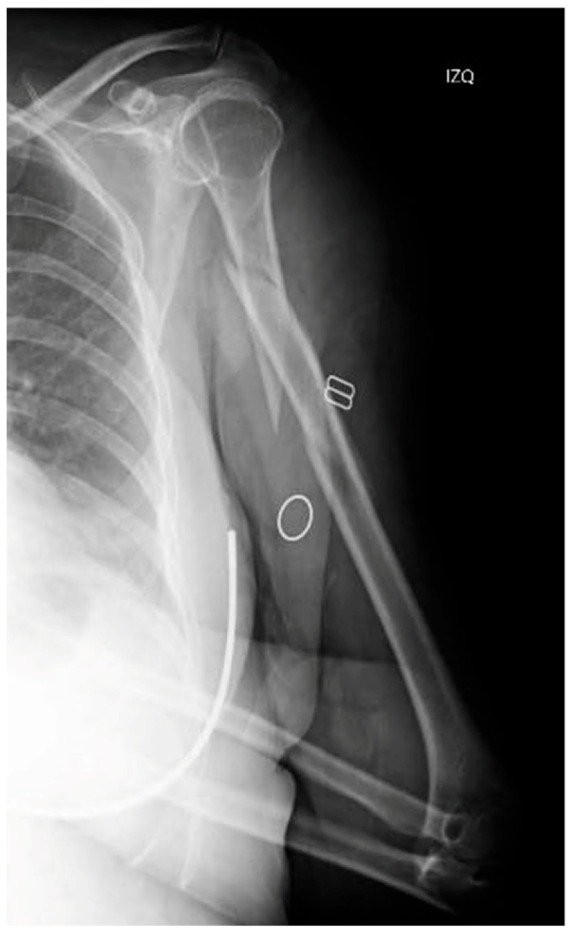
X-ray of the initial injury, showing a proximal humerus fracture extending into the diaphysis.

This is the case of a 62-year-old female who was admitted to our hospital following an accidental fall down the stairs that resulted in trauma to her (non-dominant) left upper limb. The patient had a history of ischemic heart disease, and her physical examination revealing pain, deformity and functional disability in the arm. A distal neurovascular examination detected no abnormalities. The radiological examination revealed an AO 12B2 segmental spiral fracture in the proximal third of the humeral shaft [4] (Figure 1).

**Figure 2:**
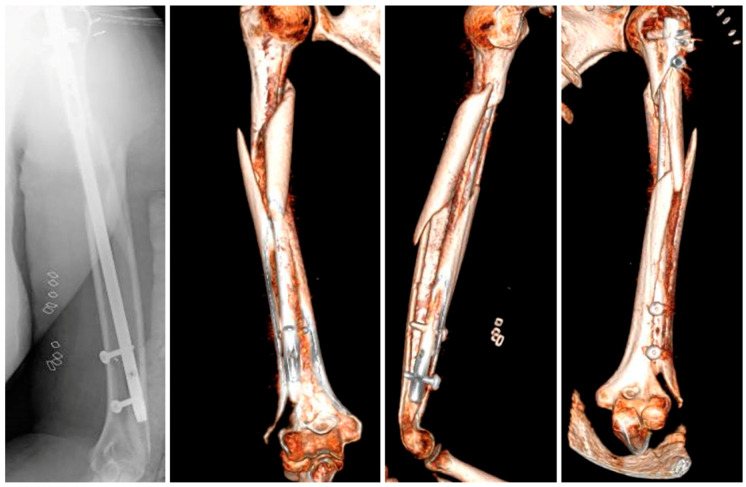
X-ray and 3D reconstruction of the peri-implant fracture.

After an initial assessment, a closed reduction was performed followed by placement of an antegrade intramedullary nail. Although there were no issues related to the diameter or curvature of the nail used, a peri-implant fracture occurred at the level of the distal interlocking screw during its insertion, as confirmed by intraoperative fluoroscopy (Figure 2). A decision was made to put an end to the procedure and perform an axial CT-scan to evaluate the morphology of the fracture.

The CT images exhibited a complex fracture originating at the lateral humeral condyle and extending sagittally towards the proximal humerus where it joined the primary spiral fracture, involving the whole of the humeral shaft. Based on this information, it was decided to perform an open reduction and internal fixation of the fracture, using a distal lateral plate combined with a proximal helical plate to ensure a stable fixation.

**Figure 3:**
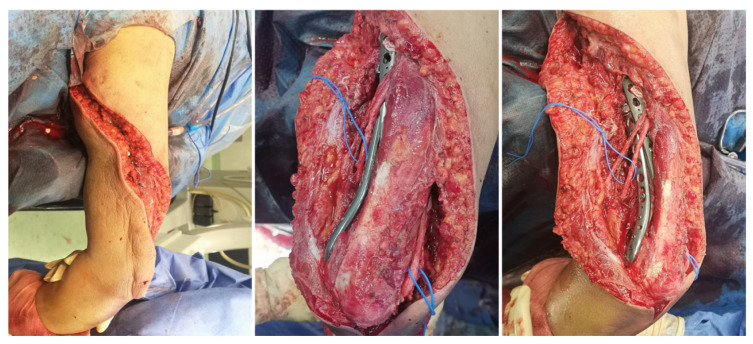
Intraoperative images showing the extended posterior elbow approach.

After removing the intramedullary implant, a rotator cuff repair was carried out at the nail’s insertion site in order to allow a speedy functional recovery. Subsequently, an extended posterior approach to the elbow was performed, proximally prolonged to the medial side, following a spiral-shaped trajectory. During the procedure, the radial and ulnar nerves were identified and dissected (Figure 3).

**Figure 4:**
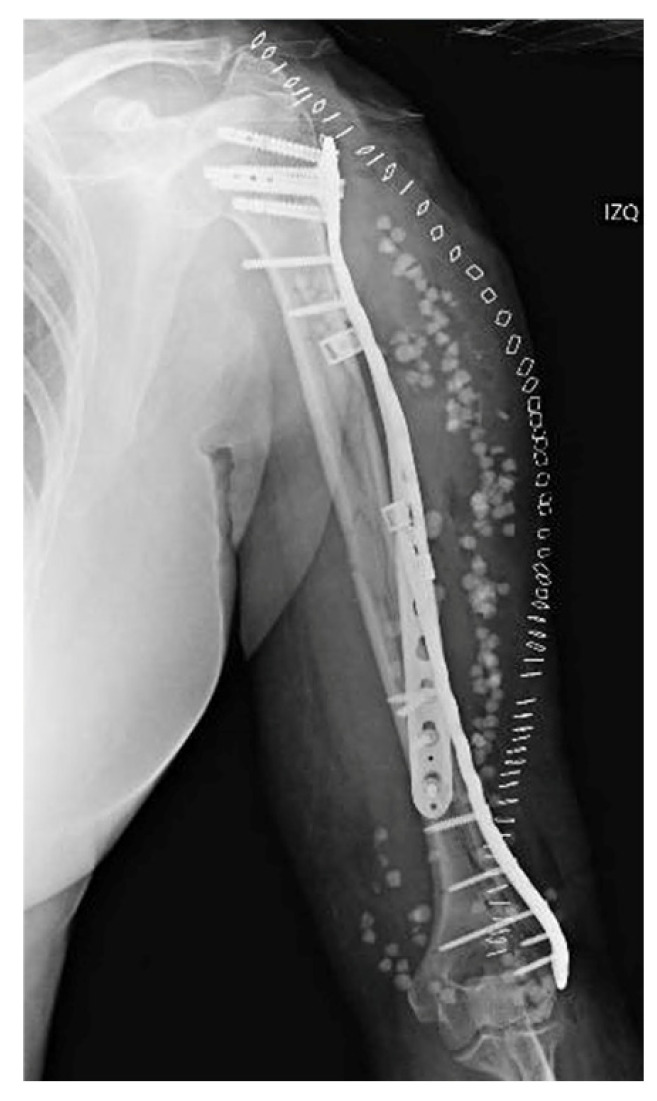
Postoperative anteroposterior view. Osteosynthesis with two precontoured helical plates and Stimulan antibiotic beads.

After reducing the fragments under fluoroscopic guidance, the two plates used to fix the fractures were supplemented by a locked extraarticular plate (LOQTEQ, aap Implantate AG, Berlin, Germany). Subsequently, and after a preliminary stabilization with K-wires, the fixation was reinforced by means of a long locked proximal humeral plate (Pantera, Toby Orthopaedics, Miami, FL, USA), which was helically contoured. The intermediate fragments were controlled using polymer cerclage bands (Ortholox, Ortolog Medical, Ankara, Turkey). Before closure, the stability of the fixation and the patient’s dynamic range of motion were checked and antibiotic-loaded calcium sulfate beads (Stimulan, Biocomposites, Staffordshire, UK) were applied as prophylaxis, as a local adjunct to systemic antibiotic therapy to enhance infection control in the context of a prolonged reoperation (Figure 4). Given the acute nature of the fracture, neither bone grafting nor postoperative electromagnetic therapy was deemed necessary. Initial immobilization was achieved by means of a Robert Jones bandage. Postoperative recovery proceeded uneventfully, with physical therapy being progressively introduced.

**Figure 5:**
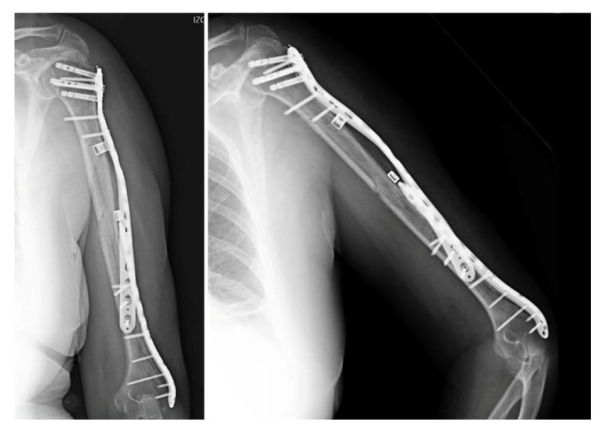
Radiographic follow-up at 6 months post-op.

Two weeks after surgery, the patient achieved 100° flexion and −15° extension, without any kind of secondary displacements being radiographically apparent. At three months, X-rays demonstrated that healing was progressing satisfactorily, with the patient exhibiting a normal range of motion. The 6-month radiographs showed complete healing of the fracture, with the physical exam revealing a full range of motion. At one year from the procedure, function was assessed by means of the Disabilities of the Arm, Shoulder and Hand (DASH) scale and the Mayo Elbow Performance Score (MEPS), in which the patient obtained scores of 86 and 75 points, respectively (Figure 5). Simultaneously, the patient was referred to Rheumatology due to a low-energy fracture and a history suggestive of bone fragility. Osteoporosis was confirmed, and treatment with calcium, vitamin D, and antiresorptive therapy was initiated.

## 3. Discussion

NPPIFs may occur in any bone with an implant. They are typically caused by the development of elasticity modulus differences between the metal of the implant and the surrounding bone, which generate stress risers that increase the risk of fracture in the area. It must be said, however, that the stresses giving rise to such fractures are usually similar in magnitude to those causing conventional fractures [1].

The main problem with NPPIFs is that they occur against the background of compromised bone stock and in the presence of an already-implanted device, which usually results in the use of complex surgical techniques selected from a reduced set of options [1]. In spite of this, the orthopedic surgeon is obliged to follow the fundamental principles of fracture management and must strive to restore the biomechanical integrity of the bone by creating a biological environment favorable to healing and employing stable mechanical constructs that facilitate the consolidation process [1]. However, information on NPPIFs is scarce and the few existing treatment algorithms refer to those occurring in the femur [1,2,3], with the few studies related to peri-implant humerus fractures referring only to periprosthetic fractures [5].

In fact, no universally accepted classification systems have been published for NPPIFs, which could explain the lack of treatment algorithms for those fractures. One of the most popular classifications is the one put forward by Egol [2]. However, this classification is by no means exhaustive and the categories proposed are far from clear cut, so much so that the case presented here could be said to represent a combination of three different categories in the classification [2]. Chan’s classification offers fewer options but includes a modifier that takes into consideration the extent to which the fracture has healed [3]. The fracture sustained by the patient in this report could be classified under Chan’s subtype C, equivalent to a two-level fracture whose recommended treatment consists of the removal of the original implant followed by fixation of the new fracture line given the greater degree of stabilization required in these cases. Regardless of the actual fracture involved, this recommendation coincides with that of other authors who advocate for the removal of the previous implant in all NPPIFs of the humerus [1].

In our case, the fracture pattern made it unfeasible to use a long intramedullary implant, and its extent precluded the use of a single plate. Given that the distal portion did not reach as far as the joint and that the degree of comminution was low, it was possible to address the case with a single lateral plate. With regard to the proximal portion of the fracture, as radial nerve involvement could entail a serious complication in plated humeral fractures [6], it was decided to helically contour the plate. A humeral helical plate has the potential to prevent damage to the radial nerve and is also able to spare the anterior portion of the deltoid [7,8]. Moreover, a helical shape has been shown to better absorb torsional stresses, a very useful feature in spiral fractures, and to provide a firmer anchorage to the bone, which reduces the risk of loosening [9].

NPPIFs are certainly infrequent but constitute a significant technical challenge for the surgeon. This case reminds us that surgeons must be cautious when selecting locked humeral nails to address diaphyseal humeral fractures; offers us guidance for resolving similar cases; and underscores the importance of performing an intraoperative fluoroscopic assessment, on the basis of which the surgeon may decide to modify the initially selected fixation technique.

## 4. Conclusions

Humeral NPPIFs are challenging and require individualized, biomechanically sound strategies. This case reinforces the importance of intraoperative assessment and careful implant selection in humeral fracture management.

## Data Availability

The original contributions presented in this study are included in the article. Further inquiries can be directed to the corresponding author.

## Abbreviations

NPPIF	Non-prosthetic peri-implant fracture
DASH	Disabilities of the Arm, Shoulder and Hand
MEPS	Mayo Elbow Performance Score
